# Poloxamer/HPMC/Carbopol-Based Thermosensitive Hydrogel Loaded with Ibuprofen for Potential Vaginal Drug Release

**DOI:** 10.3390/gels12090807

**Published:** 2026-09-03

**Authors:** Gladys Arline Politrón Zepeda, Ernesto Tinajero-Díaz, Antxon Martínez de Ilarduya, Rogelio Rodríguez Rodríguez, Gregorio Guadalupe Carbajal Arízaga, Aldo Corona Escalera, Nathaly Vasquez Martínez, Moisés Martínez Velázquez, Zaira Yunuen García Carvajal

**Affiliations:** 1 Biotecnología Médica y Farmacéutica, Centro de Investigación y Asistencia en Tecnología y Diseño del Estado de Jalisco A.C., (CIATEJ), Normalistas No. 800, Guadalajara 44270, Mexico; glpolitron_al@ciatej.edu.mx (G.A.P.Z.); alcorona_al@ciatej.edu.mx (A.C.E.); 2 Departament d’Enginyeria Química, Barcelona School of Industrial Engineering (ETSEIB), Universitat Politècnica de Catalunya, Diagonal 647, 08028 Barcelona, Spain; antxon.martinez.de.ilarduia@upc.edu; 3 Departamento de Ciencias Naturales y Exactas, Centro Universitario de los Valles (CUVALLES), Universidad de Guadalajara, Carretera Guadalajara-Ameca Km. 45.5, Ameca 46600, Mexico; rogelio.rodriguez4085@academicos.udg.mx; 4 Departamento de Química, CUCEI, Universidad de Guadalajara, Guadalajara 44430, Mexico; gregorio.carbajal@red.cucei.udg.mx; 5 Departamento de Bioquímica, Facultad de Medicina, Universidad Nacional Autónoma de México, Ciudad de México 04510, Mexico; nathaly@bq.unam.mx

**Keywords:** thermosensitive hydrogel, vaginal drug delivery, ibuprofen salt, Pluronic F127, Peppas–Sahlin model, HeLa cells

## Abstract

Vaginal drug delivery offers a critical route for local treatments but is limited by short formulation residence times. This study describes a thermosensitive in situ gel prepared by the cold-dissolution method from a ternary blend of Pluronic F127, Carbopol 940, and HPMC for localized vaginal therapy. We used ibuprofen as a model drug selected for its reported anti-inflammatory and antiproliferative activity. The hydrogels exhibited a constant gelation temperature of 28 °C and high viscosity under simulated physiological conditions; ibuprofen incorporation further reduced susceptibility to gravitational leakage. FTIR, XRD, and DSC analyses confirmed stable physical cross-linking of the polymer network and amorphous molecular dispersion of ibuprofen. Peppas–Sahlin modelling revealed a controlled, sustained release profile (>50% over 24 h) predominantly governed by Fickian diffusion (69%). The blank hydrogel exhibited high biocompatibility (>75% viability). In contrast, the ibuprofen-loaded matrix exhibited a concentration-dependent cytotoxic effect on HeLa cervical cancer cells, reducing cell viability to ~12% at the full extract concentration. Overall, this ternary hydrogel platform represents a stable, promising vehicle for sustained local administration of ibuprofen in the vaginal microenvironment.

## 1. Introduction

Vaginal drug delivery is a critical route for the treatment of local conditions, including infections, inflammation, and hormonal imbalances. This route is favoured due to its large surface area, rich vascularization, and ability to bypass first-pass hepatic metabolism [1]. The high permeability of the cervicovaginal epithelium facilitates drug absorption; however, this can be influenced by physiological factors, including pH, the mucosal barrier, the immune system, microbiota, and hormones. These factors can make it challenging to achieve optimal therapeutic concentrations at the desired site [2,3]. Consequently, the development of controlled drug-delivery systems is crucial to enhance local bioavailability, reduce systemic toxicity, and improve delivery of therapeutic agents [4].

A range of vaginal delivery platforms has been investigated, each with its own limitations. Tablets, suppositories, and conventional gels tend to be rapidly cleared by vaginal secretions [5,6,7]. Thin films are limited by their drug-loading capacity, while inserts or rings may cause local discomfort, particularly during short-course therapies [8,9]. Complex nanocarriers, such as liposomes and nanoparticles, can improve mucosal targeting, but they face challenges in translation and scale-up for broader use [10]. In contrast, in situ gelling hydrogels offer a promising alternative to overcome these limitations. These innovative formulations remain as low-viscosity liquids at room temperature and undergo a sol–gel transition, thereby simplifying administration. Upon exposure to physiological conditions, they change into a solid-like gel, enabling longer residence time and improved drug retention [11,12].

Among the polymers used to develop in situ gelling systems, poloxamers have emerged as among the most promising materials due to their reversible thermogelation and biocompatibility [13,14,15]. They are triblock copolymers composed of poly(ethylene oxide)–poly(propylene oxide)–polythylene oxide) (PEO–PPO–PEO) that self-assemble via micellization and can interact with both hydrophilic and hydrophobic drugs, thereby enabling their use as controlled-release systems [16]. In particular, Poloxamer 407 (Pluronic^®^ F127; designated herein as PF127) is approved by the Food and Drug Administration (FDA) and the European Medicines Agency (EMA) as a pharmaceutical excipient, making it attractive for the formulation of vaginal delivery systems [17].

Nevertheless, pure poloxamer-based gels often exhibit weak mechanical strength and rapid erosion [18]. To overcome these limitations, incorporating secondary polymers, such as Carbopol 940 and hydroxypropyl methylcellulose (HPMC), can significantly enhance mucoadhesive strength, mechanical stability, and drug release control, and stabilize the drug through synergistic matrix interactions [19].

Carbopol^®^ 940 (Lubrizol Corp., Wickliffe, OH, USA; referred to as CP940) is a synthetic homopolymer of polyacrylic acid crosslinked with allyl sucrose or allyl pentaerythritol, polymerized in a cosolvent system [20]. CP940 is widely recognized for forming pH-sensitive hydrogels that rapidly develop viscosity and mechanical strength upon neutralization. These properties facilitate sustained drug release and stable therapeutic levels over the desired period [19].

HPMC is a semi-synthetic, non-ionic cellulose derivative that has gained attention as a low-cost, renewable alternative to synthetic polymers [21]. Widely used as a pharmaceutical excipient, it forms stable hydrogel films and matrices that enable fine-tuning of drug release kinetics [19].

Beyond designing the delivery platform, the choice of active pharmaceutical ingredients is equally critical for achieving the desired therapeutic outcome. Vaginal inflammation plays a pivotal role in the pathophysiology of multiple gynaecological diseases [22], including recurrent infections [23], epithelial alterations [24], and neoplastic processes [25], in which sustained production of pro-inflammatory mediators drives tissue damage and pathology [26]. Chronic inflammation is also a crucial driver of cervical carcinogenesis, contributing to increased prostaglandin production, cell proliferation, angiogenesis, and resistance to apoptosis [27].

Ibuprofen, a non-steroidal anti-inflammatory drug (NSAID), is commonly employed to manage pain and inflammation. Additionally, it represents an attractive candidate for localized vaginal delivery due to its dual anti-inflammatory and anticancer properties. Its primary mechanism of action involves inhibiting cyclooxygenase (COX) enzymes, thereby reducing the synthesis of pro-inflammatory prostaglandins derived from arachidonic acid [28,29,30]. In addition to COX inhibition, ibuprofen modulates inflammation-related tumorigenic pathways and demonstrates antiproliferative effects on HeLa cervical cancer cells by decreasing viability and inducing apoptosis [31]. Based on this dual mechanism, ibuprofen has been proposed in the literature as a candidate for combined local anti-inflammatory and antiproliferative therapy [27], which motivated its selection for the present delivery platform. Furthermore, local vaginal administration may enhance drug concentrations at the target site while minimizing the gastrointestinal, renal, and cardiovascular adverse effects commonly associated with oral or parenteral NSAID therapy [31,32].

Ibuprofen has been used clinically for the topical treatment of vulvovaginal inflammation, particularly as ibuprofen isobutanolammonium vaginal solutions (Ginenorm^®^). However, these liquid formulations have a limited residence time in the vaginal cavity [33]. Therefore, thermosensitive hydrogels represent a promising strategy to prolong drug retention and enhance local bioavailability [34].

Binary systems such as poloxamer/HPMC [35] and poloxamer/Carbopol [36] have been evaluated as in situ-forming thermosensitive hydrogels, and the ternary F127/HPMC/CP940 combination has also demonstrated potential for an in situ gel system for the sustained release of anti-tuberculosis drugs [19]. However, the ternary combination remains unexplored for vaginal drug delivery. In this context, integrating these polymers offers a promising approach to developing in situ gelling systems with optimized thermoresponsiveness, enhanced mucoadhesion, and controlled release.

The purpose of this work was to characterize a thermosensitive hydrogel platform based on a ternary blend of PF127, CP940, and HPMC, tailored for localized vaginal administration of ibuprofen. Ibuprofen was selected as a model drug to assess the system’s performance. The study includes comprehensive physicochemical characterization, including evaluation of pH, viscosity, chemical interactions, and structural information obtained by Fourier Transform Infrared Spectroscopy (FTIR) and X-ray diffraction (XRD). The thermal profile was also evaluated by differential scanning calorimetry and thermogravimetric analysis (DSC/TGA), along with the swelling capacity of the formulations. We also assessed critical functional parameters that determine clinical applicability were assessed, such as gelation time and temperature, gel persistence, spreadability, and adhesive properties. We analyzed the in vitro drug release profile in simulated vaginal fluid (SVF) using the Peppas–Sahlin kinetic model to decouple the release mechanisms. Finally, we evaluated the biological activity of the formulations in a two-dimensional (2D) HeLa cancer cell culture model.

To the best of our knowledge, this is the first report to adapt the PF127/HPMC/CP940 system to incorporate ibuprofen within the matrix, highlighting its potential as a vaginal delivery system.

## 2. Results and Discussion

The following sections detail the systematic evaluation of the developed hydrogel platform as a potential vehicle for localized vaginal drug delivery. First, we discuss baseline quality-control metrics for the formulations. Building on this foundation, we assessed the functional and thermoresponsive behaviours, including gelation time and temperature, gel persistence, spreadability, adhesiveness, and leakage resistance, all evaluated under simulated physiological conditions. We then discuss the structural integration of the ternary blend, as evidenced by spectroscopic, thermal, and crystallographic analyses. Finally, we assessed the overall performance of the hydrogels by correlating sustained drug-release kinetics in SVF with in vitro cytotoxic efficacy against HeLa cervical cancer cells.

### 2.1. Preparation of Hydrogel

**Figure 1:**
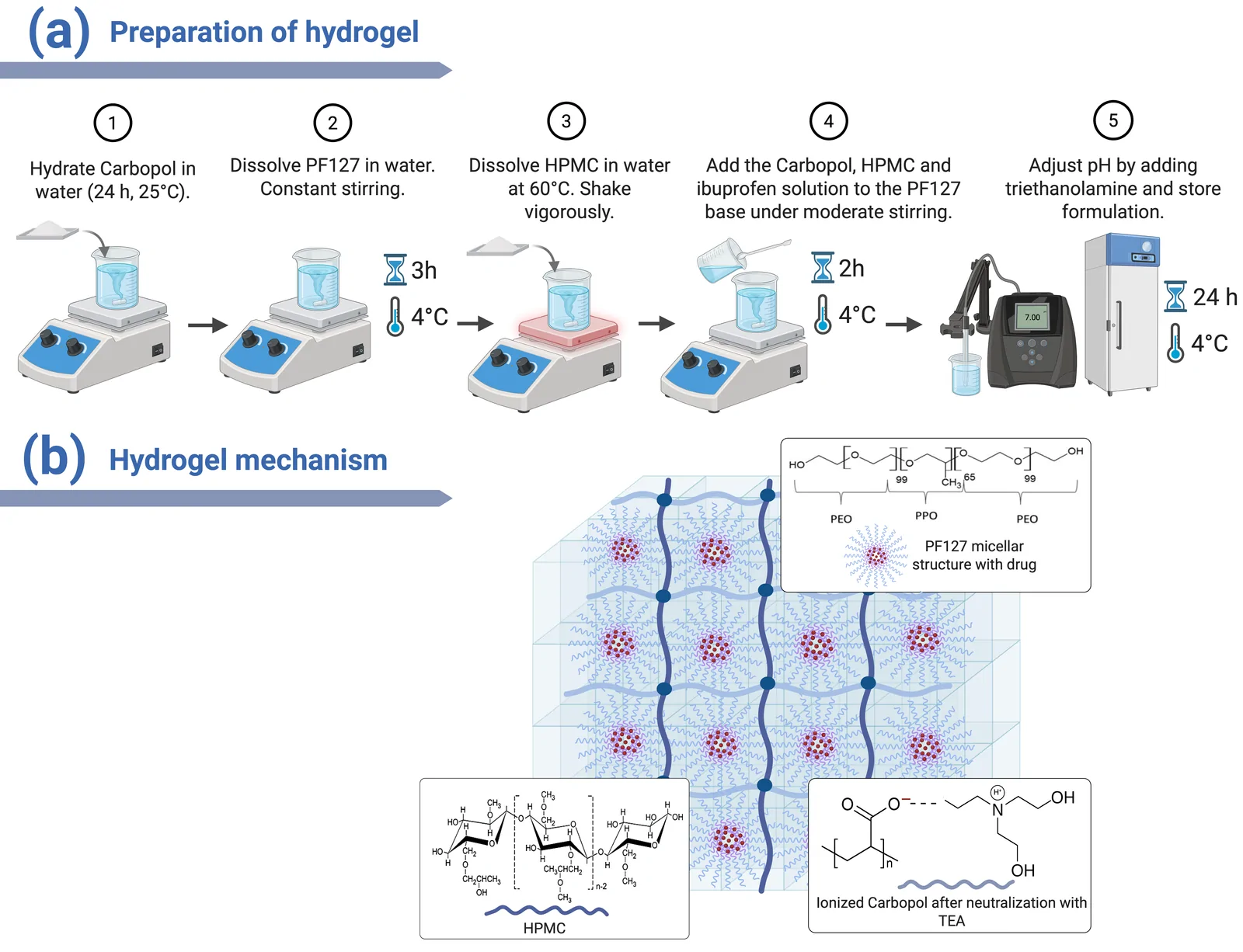
Preparation and proposed microstructural network of the thermosensitive hydrogel. (**a**) Schematic workflow of the formulation process utilizing the cold dissolution method, from polymer hydration to final pH adjustment with TEA. (**b**) Conceptual illustration of the ternary polymeric interactions, showing drug-loaded PF127 micellar structures packed within the entangled crosslinked matrix of HPMC and TEA-neutralized CP940. Figure created by the authors using BioRender.com. Agreement number: CR29WJHOQO.

Ibuprofen-free (H1) and ibuprofen-loaded (H1IBU) hydrogels were prepared using the cold method, selected to preserve the thermosensitive properties of PF127, facilitate the homogeneous incorporation of ibuprofen, and provide a straightforward preparation route compatible with autoclave sterilization (Figure 1a) [37].

We defined the selected polymer composition based on a previously published mixture design study by our research group [38]. In that study, we prepared nine formulations by systematically varying the concentrations of PF127 (15–17%, *w*/*w*) and HPMC (0.1–0.3%, *w*/*w*), while maintaining CP940 at 0.1% (*w*/*w*). The optimized hydrogel consisted of 17% (*w*/*w*) PF127, 0.1% (*w*/*w*) CP940, 0.3% (*w*/*w*) HPMC, and 0.23% (*w*/*w*) ibuprofen, selected to balance pre-administration fluidity and post-administration viscosity for handling and prolonged residence at the administration site. This design criterion is consistent with previous reports highlighting that the rheological properties of vaginal gels should balance ease of administration, spreading, leakage resistance, and residence time [39].

The cold method yielded homogeneous hydrogels without visible phase separation, sedimentation, or drug crystallization. At 4 °C, both H1 and H1IBU exhibited low viscosity, facilitating liquid handling before thermal gelation. No changes in visual appearance or phase stability were observed following autoclave sterilization or during refrigerated storage (4 °C) for up to 1 month. This indicates adequate baseline physicochemical stability, consistent with the storage windows reported as acceptable for similar poloxamer-based hydrogels [35,40]. This behaviour is attributed to the extensive hydration of the hydrophilic PEO chains of PF127 at low temperatures, which prevents premature aggregation [41].

Moreover, in aqueous media, PF127 can self-assemble into amphiphilic micelles comprising a hydrophobic PPO core and a hydrophilic PEO shell. Micellization represents the first step in gelation, as the physical packaging of the micellar structures is fundamental to the formation of the three-dimensional gel network. The gelling phenomenon is reversible and is associated with a sol–gel transition temperature [42]. PF127 has been successfully employed as a carrier for ibuprofen due to its self-assembling micellar behaviours [43]. Its amphiphilic structure is expected to enable ibuprofen to partition into the hydrophobic domains of PPO, a mechanism that has been reported to enhance drug solubilization and promote uniform dispersion within the polymer matrix [35]. Furthermore, low concentrations of CP940 are reported to reinforce the polymeric network and improve the structural stability of thermosensitive poloxamer-based hydrogels without compromising their thermoresponsive behaviour [15,44]. Meanwhile, triethanolamine (TEA) was incorporated to neutralize the carboxylic groups of CP940, thereby increasing ionization and generating electrostatic repulsion between Carbopol polymer chains [45]. This process induces network expansion, increases viscosity, and enhances hydrogel stability. The ionized structure of Carbopol polymers is further reported to support mucoadhesion via hydrogen-bond interactions with mucin glycoproteins, prolonging residence time and improving drug retention at the vaginal mucosa (Figure 1b) [45,46].

Autoclave sterilization is a suitable method for poloxamer-based hydrogels. Beard et al. (2021) demonstrated that autoclaving at 121 °C for 20 min preserved the rheological behaviour and drug-release performance of PF127 hydrogels, despite minor reductions in water content and gelation temperature, supporting the use of autoclaving for H1 and H1IBU hydrogels [47].

The successful integration of a thermoresponsive polymer (PF127), a mucoadhesive polymer (CP940), and a viscosity-enhancing polymer (HPMC) resulted in a stable hydrogel platform with characteristics suitable for vaginal administration [19,34,35].

### 2.2. Physicochemical Characterization and Performance Parameters

Physicochemical and functional characterization is essential to ensure the structural integrity, stability, and mucosal compatibility of hydrogels designed for vaginal delivery [12,48,49]. Accordingly, we evaluated H1 and H1IBU across key performance parameters. We autoclaved the hydrogels to ensure all evaluations were performed on the final formulation intended for vaginal administration. Steam sterilization is a widely used terminal sterilization method in the healthcare industry because it effectively reduces microbial contamination [50].

#### 2.2.1. pH

Formulation pH is a critical parameter in the design of vaginal drug delivery systems, as it directly influences matrix stability, mucosal tolerance, and drug solubility [49].

Neutralization of carboxylic groups in CP940 is required to induce ionization, thereby generating electrostatic repulsion between adjacent polymer chains that promotes network expansion and increased viscosity [51,52]. Consequently, formulations were initially adjusted to pH 7.0 to achieve optimal gelation.

Freshly prepared, sterilized hydrogels exhibited pH values of 7.14 ± 0.11 for H1 and 7.34 ± 0.11 for H1IBU (Table 1). After one month of storage at 4 °C, both formulations showed only a slight decrease to approximately 6.8, suggesting adequate pH stability consistent with the reported ranges for similar Carbopol polymers and poloxamer systems [53,54].

Although the healthy vaginal environment is acidic (pH 3.8–4.5), a near-neutral pH was necessary to ensure CP940 ionization. Because the applied hydrogel volume is small relative to the buffering capacity and the continuous lactic acid production of the vaginal microenvironment, local pH elevations are expected to be transient and naturally neutralized by fluid dilution. However, this study did not monitor pH-drift kinetics upon dilution or in vivo pH recovery, which are essential requirements before clinical translation.

Finally, to evaluate functional performance under physiologically relevant acidic conditions, all subsequent evaluation—including swelling, in vitro adhesion, drug solubility, and release kinetics—was conducted in SVF (pH 4.6).

#### 2.2.2. Swelling Profile

The swelling capacity of hydrogels directly influences their mucoadhesive performance, mucosal residence time, and drug-release behaviour, making it a key parameter in the formulation design of vaginal drug-delivery systems [55,56,57].

**Figure 2:**
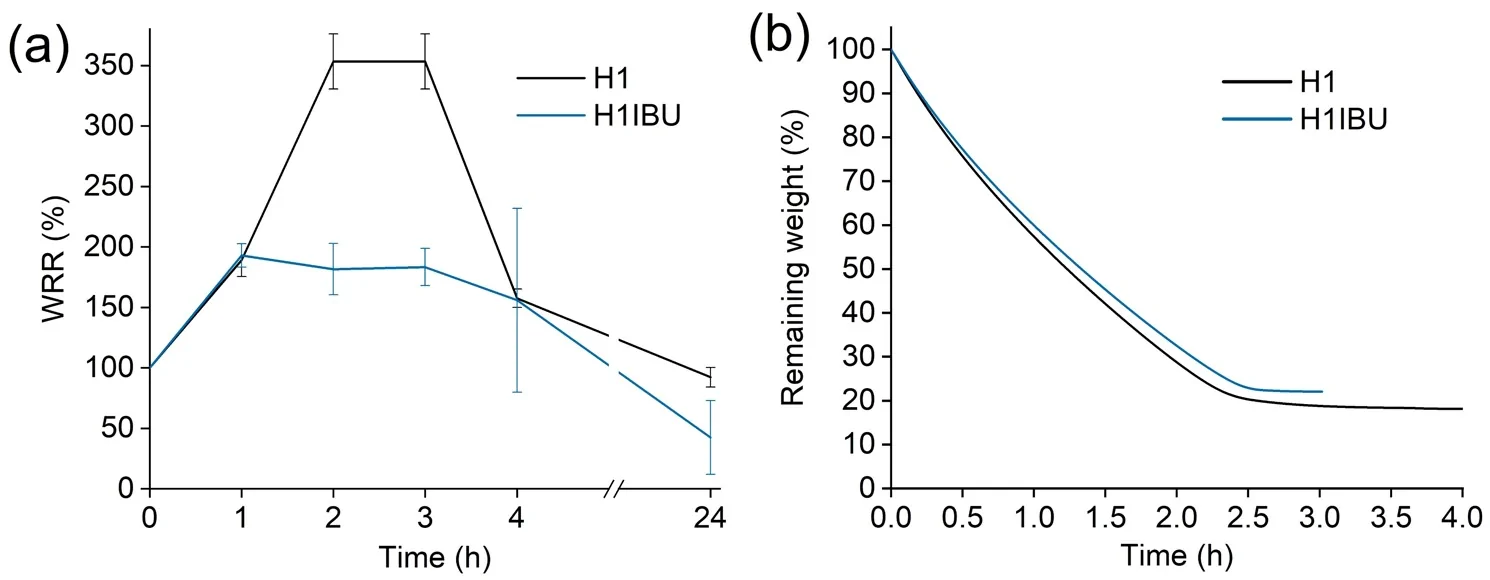
Water retention and dehydration kinetics of H1 and H1IBU hydrogels. (**a**) Water retention ratio (%WRR) curves and (**b**) isothermal water loss profiles at 37 °C for drug-free (H1) and ibuprofen-loaded (H1IBU) hydrogels. Statistically significant differences were determined using two-way ANOVA with a Tukey post hoc test (*p* < 0.05). Data are presented as mean ± SD (*n* = 3).

H1 exhibited a swelling ratio (SR) of 2.58 ± 0.16 and a water retention ratio (%WRR) of approximately 353% after 3 h, indicating high capacity to absorb and retain aqueous media (Table 1, Figure 2a). This behaviour is consistent with reports that the hydrophilic nature of CP940, PF127 and HPMC promotes water uptake via hydrogen bonding, forming a hydrated three-dimensional network [58,59]. Such hydration properties are expected to support intimate contact with the mucosal surface and enhance mucoadhesion [34,52]. Furthermore, matrix hydration can influence drug diffusion kinetics by promoting polymer chain relaxation and network permeability [58,60].

In contrast, the H1IBU formulation displayed a substantially lower SR (0.75 ± 0.03) and a reduced water-retention capacity, with %WRR decreasing from 182% at 3 h to 41% after 24 h. This reduction may be attributed to the hydrophobic nature of ibuprofen (pKa = 4.5, logP ≈ 3.5) [61], which can reduce the overall affinity of the polymeric network for water and limit water penetration into the hydrogel matrix [62,63]. Moreover, previous studies have reported that, in poloxamer-based systems, hydrophobic molecules can preferentially partition into the PPO micellar cores, potentially modifying the polymer network and decreasing the availability of hydrophilic regions for water accommodation [42,64]. This profile is also consistent with an interplay between restricted water uptake, polymer relaxation, and gradual matrix erosion during prolonged incubation [65]. Because the present study did not include structural or thermodynamic analyses, these mechanisms remain speculative interpretations of the observed swelling behaviour and should be investigated in future studies.

#### 2.2.3. Water Loss

We monitored isothermal mass loss at 37 °C to evaluate the hydrogel dehydration kinetics under simulated physiological conditions (Figure 2b). H1 and H1IBU showed similar profiles, with rapid water loss during the first 2.5 h followed by a stable plateau, consistent with the biphasic dehydration kinetics reported for hydrogel systems [66,67,68]. The remaining mass stabilized at approximately 20%, consistent with the formulation’s total solid fraction and indicating an initial solvent content of 80%. The lack of significant differences between H1 and H1IBU suggests that incorporating ibuprofen does not alter the matrix’s water-retention capacity. Dehydration dynamics are therefore governed by the PF127, HPMC, and CP940 network, consistent with the water-interacting roles previously described for these polymers [42,58].

#### 2.2.4. Viscosity Measurements

Viscosity was evaluated at 25 °C (20 rpm) and 37 °C (0.2 rpm) to simulate application dynamics and resting conditions within the vaginal cavity, respectively. The higher shear rate applied was selected to simulate the extrusion forces reported for administration via a vaginal applicator, providing insight into the formulation’s spreadability [69]. Conversely, the low shear rate at 37 °C was selected to approximate the resting state of a stationary hydrogel depot after administration [18].

Before sterilization, viscosity measurements performed under simulated resting conditions (37 °C, 0.2 rpm) showed values of 2,295,333 ± 46,971 mPa·s for H1 and 2,552,333 ± 348,491 mPa·s for H1IBU. Although autoclaving caused minor changes in viscosity (Table 1), the thermoresponsive transition was preserved in both formulations, consistent with the autoclave stability described in Section 2.1.

Post-sterilization measurements confirmed that temperature-dependent rheological changes were primarily governed by PF127 (Table 1). At 25 °C, H1 and H1IBU exhibited low viscosities of 150,833 ± 41,135 mPa·s and 143,233 ± 4485 mPa·s (*p* > 0.05), respectively. Viscosities in this range are reported to facilitate comfortable handling and extrusion before gelation [14,69]. At 37 °C, a marked viscosity increase was recorded, reaching 2,603,333 ± 223,681 mPa·s for H1 and 2,112,333 ± 270,601 mPa·s for H1IBU. The phase transition is generally attributed to temperature-induced micellization and dense micellar packing within Carbopol/HPMC systems [42]. Although ibuprofen slightly decreased maximum viscosity at 37 °C—a phenomenon described in the literature to arise from hydrophobic interactions with PPO domains that alter micellar packing [70,71,72,73]—our experimental findings show that H1IBU maintains high viscosity at rest. This behaviour supports the system’s potential for vaginal administration, as it enables easy application at room temperature and rapid in situ gelation, which may minimize leakage and prolong mucosal retention.

#### 2.2.5. Sol–Gel Transition Characterization

The hydrogels exhibited a constant gelation temperature of 28 °C across all conditions tested, confirming a rapid in situ transition to a solid-like state at physiological temperature regardless of drug loading or pH (Table 2). Both drug incorporation and medium pH significantly influenced gelation time, with H1IBU displaying the fastest transition at neutral pH (16.66 ± 2.88 s). Although this study did not directly investigate the underlying molecular mechanism, ibuprofen’s hydrophobic nature may favour interaction with the PPO domains of PF127. This promotes micellization and intermicellar packing, thereby accelerating the sol–gel transition kinetics [74]. Based on prior literature, this accelerated micellar organization is hypothesized to result from increased ionic strength and drug-polymer interactions, which may reduce the risk of formulation leakage [75,76].

Spreadability remained unaffected by ibuprofen incorporation, as both formulations maintained comparable spread diameters (14 mm ± 1.73 mm), a value considered favourable for administration comfort in previous reports [77,78]. However, matrix persistence diverged significantly at pH 4.6: H1 showed greater stability (178.5 ± 44.50 min) than H1IBU (76.67 ± 17.55 min). While our experimental findings document reduced persistence, the literature on poloxamer networks suggests that although ibuprofen may promote initial entanglement, it could interfere with long-term physical crosslinking density [34,41,77]. Finally, statistical analysis indicated that the reduction in gelation time observed upon neutralization is primarily driven by pH-induced changes in the network rather than by the drug’s presence. These results highlight how formulation pH influences gelation kinetics and matrix persistence.

#### 2.2.6. Leakage Assay

We assessed retention at the application site using an in vitro leakage assay under simulated vaginal conditions (SVF, pH 4.6, 90° inclination). A Student’s *t*-test revealed a significant difference (*p* < 0.001) between H1 and H1IBU, indicating that matrix composition directly influences gel cohesion under gravitational stress. H1 showed greater susceptibility to flow, with displacement of 51.67 ± 5.77 mm. Conversely, ibuprofen incorporation enhanced matrix cohesion, reducing displacement to 10.33 ± 0.58 mm (~80% reduction in mobility). In line with previously reported mechanisms, this stabilization effect can be attributed to additional intermolecular interactions between ibuprofen and the polymeric network [79]. From a clinical perspective, improved resistance to leakage is desirable, as it may support prolonged mucosal retention and localized drug action [78,80]. This assay provides a simple baseline for comparing formulation resistance to gravity and dilution; however, it operates under static conditions. Further evaluation using dynamic or ex vivo models will be required to confirm these retention properties in the presence of continuous vaginal fluid turnover.

#### 2.2.7. In Vitro Adhesion

The adhesion analysis showed that H1 had a significantly longer retention time (24 ± 5.29 min) than H1IBU (8.77 ± 1.08 min), under continuous shear stress (50 rpm). While incorporating ibuprofen enhances bulk matrix properties such as viscosity and leakage resistance, the reduced adhesion time suggests a compromise in interfacial flexibility, potentially attributable to saturation of network sites available for mucosal interaction, or increased network rigidity. In contrast, the literature suggests that the greater water retention capacity of H1 could promote interfacial hydration, thereby facilitating polymer-chain rearrangement and interaction with the mucosal surface [81,82]. These findings highlight the importance of balancing drug-induced structural reinforcement with the interfacial adaptability required to support prolonged mucosal residence [78]. Nevertheless, because these preliminary findings are based on simplified in vitro biomimetic models, future investigations need further ex vivo and in vivo mucoadhesion evaluations to establish definitive residence times and confirm tissue retention under physiological conditions.

### 2.3. FTIR

**Figure 3:**
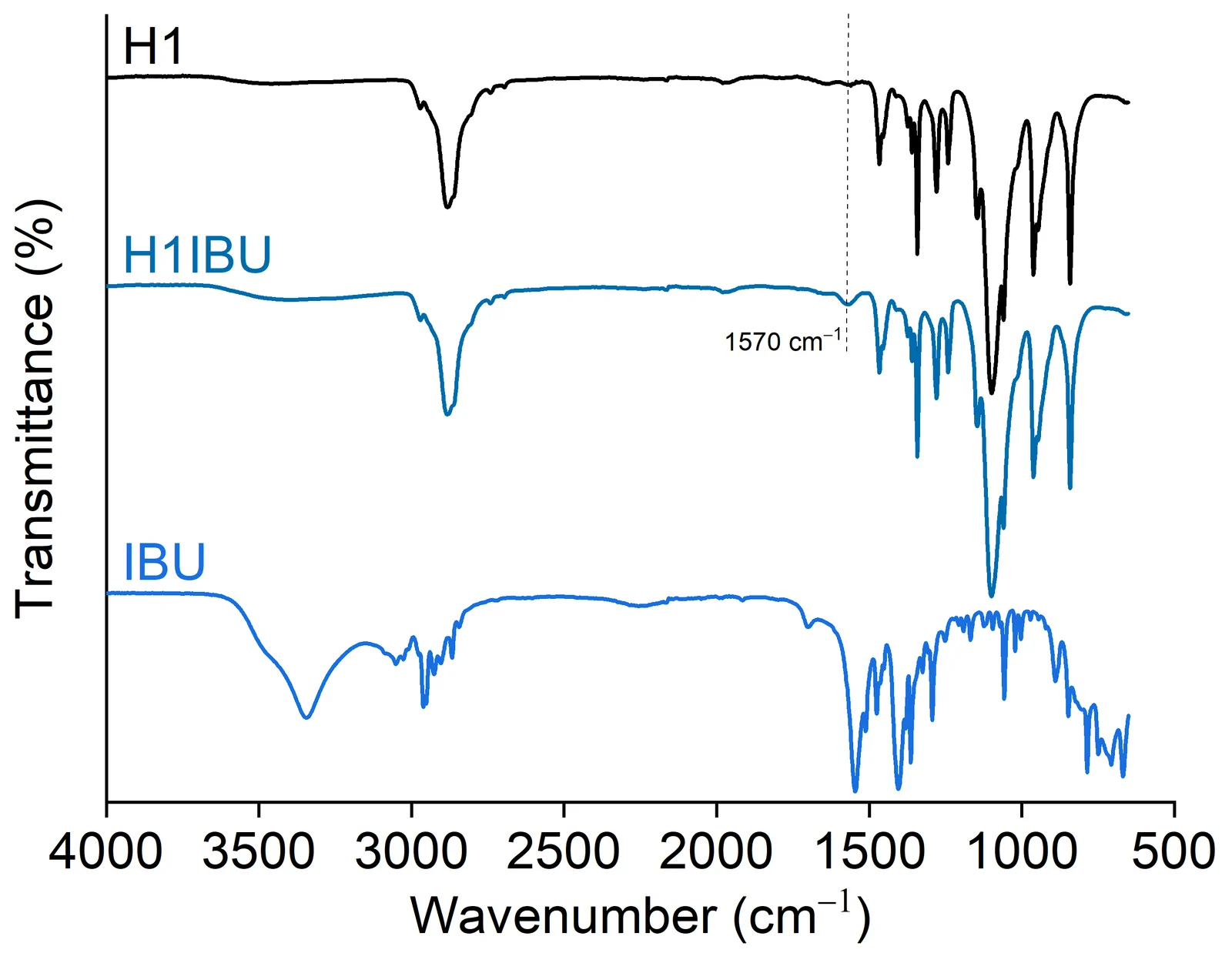
FTIR spectra of sterilized drug-free hydrogel (H1), ibuprofen-loaded hydrogel (H1IBU) and pure ibuprofen (IBU). The characteristic band at 1570 cm^−1^ in the H1IBU spectrum confirms successful incorporation of the drug within the polymeric matrix.

FTIR spectroscopy was used to evaluate the chemical stability and physical interaction profile within the hydrogel network (Figure 3). Comparison with the individual precursor spectra (PF127, HPMC, and CP940; Figure S1) showed that the H1 hydrogel retained the characteristic CH_2_ (2880–2900 cm^−1^) and C–O–C (1100 cm^−1^) bands of PF127, with no evidence of covalent modification, confirming that matrix formation arises from a physical polymer blend [19,30]. Spectral invariance at 1100 cm^−1^ and 1700 cm^−1^ between H1 and H1IBU hydrogels indicates that ibuprofen incorporation does not alter the polymeric backbone. Ibuprofen incorporation was confirmed by the appearance of a carboxylate stretching band at 1570 cm^−1^ in the H1IBU spectrum, a spectral feature reported in the literature to reflect the ionized state of the drug and its interaction with the matrix via hydrogen bonding and electrostatic forces [32]. Notably, this band was detected after autoclave sterilization, confirming that ibuprofen incorporation was retained despite thermal processing. Thus, the FTIR analysis indicates that the hydrogel forms a stable, physically cross-linked system in which ibuprofen is incorporated without disrupting the polymer network.

### 2.4. Thermal Analysis

We evaluated the thermal stability and decomposition profiles of H1, H1IBU, and their individual precursor polymers using TGA. Table 3 summarizes the characteristic thermal parameters, and the corresponding thermograms are provided in the Supplementary Materials (Figure S2).

TGA results indicated that H1 exhibits higher thermal stability than its individual precursors, with an initial degradation temperature (T_d_) of 370 °C (Figure S2a). Consistent with the published literature on ternary hydrogels, this enhanced stability is attributed to the synergistic physical cross-linking of the ternary polymer network [10,19]. Although incorporating ibuprofen slightly lowered the initial T_d_ to 357 °C due to the drug’s inherent thermolability, it increased the maximum decomposition temperature (^max^T_d_) to 403 °C (Figure S2b–d). This shift suggests that drug-polymer molecular interactions reinforce the matrix against thermal stress, a stabilizing mechanism previously reported for similar platforms [83,84]. Furthermore, our experimental thermograms showed no characteristic ibuprofen thermal signals at low temperatures, suggesting successful molecular dispersion and protection of the drug within the polymeric network. These findings support the structural stability of H1IBU under processing conditions.

We evaluated complementary thermal transitions by DSC. Figure S3 and Table 3 summarize the DSC thermograms and corresponding parameters, respectively. The experimental curves showed the persistence of the endothermic peak for PF127 (50–60 °C) in H1, confirming thermodynamic compatibility among the matrix components. Literature attributes the slight widening of this peak to intermolecular interactions—such as hydrogen bonding—with the HPMC, which reinforces the polymer network without impairing its in situ gelation capacity [85,86].

Importantly, the disappearance of the characteristic melting peak of ibuprofen (65 °C) in H1IBU indicates the transition of the drug from its crystalline state to an amorphous molecular dispersion within the matrix. As established in previous reports, overcoming the energy constraints imposed by the crystal network is biologically advantageous, as it favors a more uniform release and enhanced potential bioavailability in the vaginal microenvironment [16,87,88]. Finally, the absence of new thermal events following drug incorporation suggests the formation of a physicochemically stable system, which—as reported in previous studies—supports the system’s potential to prevent active substance recrystallization while preserving the thermoresponsive functionality essential for its application [89,90].

### 2.5. X-Ray Diffraction

**Figure 4:**
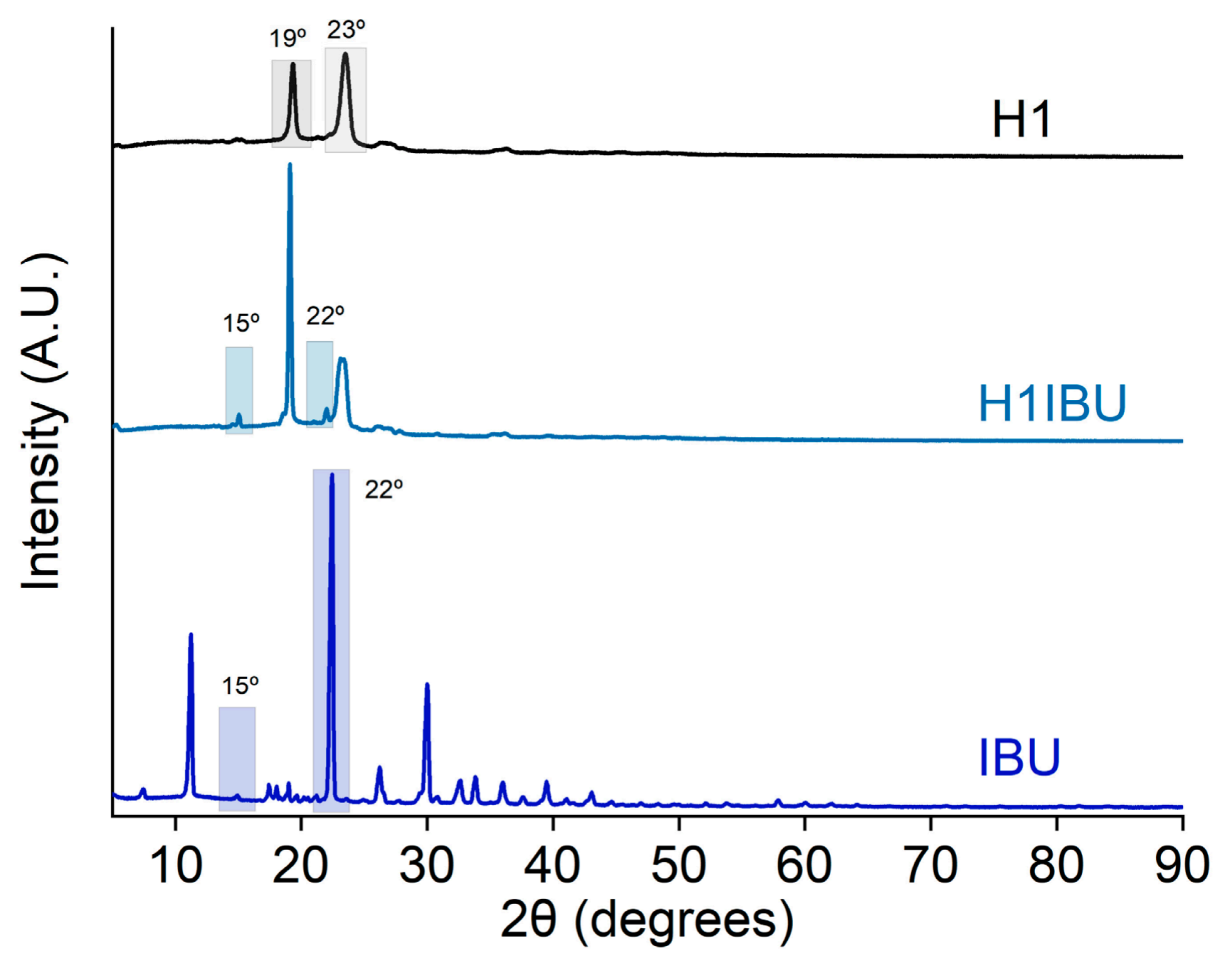
X-ray diffraction patterns of drug-free hydrogel (H1), ibuprofen-loaded hydrogel (H1IBU), and pure ibuprofen (IBU). Highlighted bands indicate main diffraction reflections (2θ) associated with the hydrogel matrix (19° and 23°) and crystalline drug (15° and 22°).

XRD-based structural characterization supported the transition of ibuprofen from a crystalline to an amorphous or low-crystallinity state, complementing the calorimetric findings. While the pure ibuprofen exhibited characteristic crystalline diffraction peaks at 2θ values of 15°, 22.3°, and 30°, these reflections were completely absent in the diffractogram of the H1IBU (Figure 4). This observation suggests a marked reduction in ibuprofen crystallinity within the polymeric matrix. The persistence of the characteristic reflections of the polymer matrix (19.2° and 23.4°), together with a slight shift towards 22.2°, suggests drug incorporation. In agreement with the literature, this peak shift can be attributed to a distortion in the packaging of PEO chains in PF127 induced by physical drug–polymer interactions [91,92,93,94]. From a biopharmaceutical perspective, this transformation is advantageous, as established in published reports, as it lowers the energy barrier to the crystal network, thereby facilitating more efficient dissolution in vaginal fluid [32,95,96]. Collectively, the XRD data suggest that the ternary matrix serves as a molecular stabilization system, preserving matrix integrity and optimizing the physical state of the active ingredient, thereby supporting its prospective therapeutic potential for local delivery.

### 2.6. Drug Release Profile

**Figure 5:**
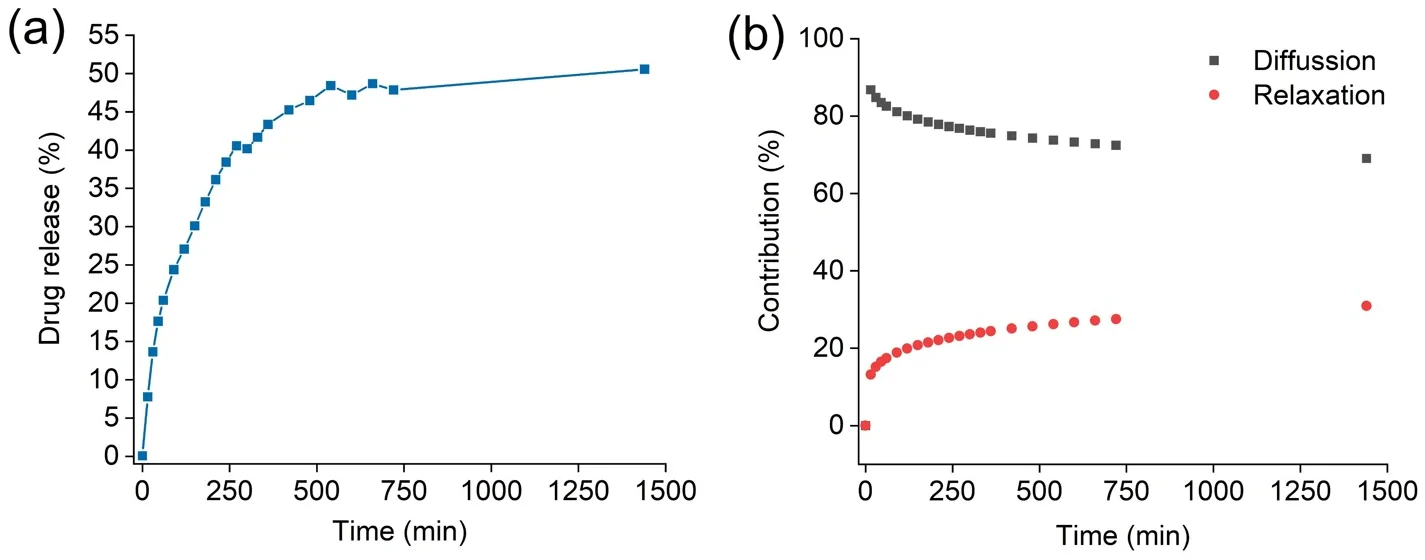
In vitro drug release kinetics and mechanistic analysis of the H1IBU hydrogel. (**a**) Cumulative ibuprofen release profile as a function of time. (**b**) Peppas–Sahlin model analysis showing the percentage contribution of Fickian diffusion and polymeric relaxation to the overall drug-release mechanism.

We evaluated the in vitro controlled release of ibuprofen from H1IBU. The release pattern depends on multiple factors, including hydrogel morphology, drug-binding affinity, and polymer hydration rate [97]. Expressed as a percentage of initial theoretical loading—a valid approximation given direct incorporation without washing steps—the cumulative release profile exhibited sustained kinetics typical of a controlled-release hydrogel (Figure 5a), consistent with the behaviour typically reported for controlled-release polymeric systems [98].

Amorphous drug dispersion (supported by DSC and XRD) and an ionized fraction of 55.73% (pH 4.5) are expected to favour high drug availability in SVF [19,99,100]. These kinetic parameters are consistent with the sustained-release profile observed experimentally, in which more than 50% of the incorporated ibuprofen was released over 24 h without an initial burst. Ibuprofen retention within the PF127/CP940/HPMC hydrogel may be associated with preferential partitioning into the hydrophobic domains of PF127 micelles, along with restricted diffusion within the three-dimensional polymeric network. Weak intermolecular interactions, such as hydrogen bonding, may also contribute to drug stabilization within the matrix. These proposed mechanisms are consistent with previous reports but were not directly evaluated in the present study [98,101,102].

Experimental release data were fitted to the Peppas–Sahlin model via non-linear regression using the Generalized Reduced Gradient (GRG) algorithm in Microsoft Excel Solver [103] (Figure 5b, Table 4), a widely used model for describing drug release from swellable polymeric matrices by decoupling the relative contributions of Fickian diffusion (*k*1) and polymer relaxation/erosion (*k*2). According to the model, Fickian diffusion accounted for 69.05% of the cumulative drug release. In comparison, polymer relaxation accounted for 30.95%, suggesting that diffusion was the predominant contributor to drug transport under the experimental conditions. The diffusional rate constant (*k*1 = 0.0748 min^−m^) was substantially higher than the relaxation rate constant (*k*2 = 0.0061 min^−2m^), yielding a *k*1/*k*2 ratio of 12.30 and a diffusional exponent (m) of 0.2364 (SSE = 2.7258). Within the Peppas–Sahlin model, these parameters are consistent with a diffusion-dominated release profile.

However, the Peppas–Sahlin model provides only a phenomenological description of the experimental release profile, rather than direct experimental evidence of the underlying molecular transport mechanisms. Consequently, interpret the diffusion prevalence derived from the mathematical fit with caution, and complementary structural and mechanistic studies are needed to elucidate the release process [104].

Hirun et al. (2024) reported a similar diffusion-controlled release profile for ibuprofen-loaded thermosensitive poloxamer micellar gels; using the Korsmeyer–Peppas model, they likewise identified Fickian diffusion as the predominant release mechanism [74]. Because the release profile in the present study did not reach a clear plateau after 24 h, further ibuprofen release would be expected over longer periods; however, the extent and rate of any subsequent release were not experimentally evaluated, and no conclusions regarding the ultimate extent of drug release can be drawn from the present study.

Taken together, these kinetic findings support the macroscopically observed behaviour of the formulation. H1IBU achieves a controlled, sustained release exceeding 50% over 24 h while avoiding an undesirable initial burst. This predictable transport profile suggests that the intermolecular interactions within the interconnected PF127, CP940, and HPMC network successfully constitute an efficient and reliable reservoir for prolonged localized vaginal administration, consistent with the sustained-release performance reported for similar ternary polymeric matrices [105,106,107].

### 2.7. Biological Assays

#### 2.7.1. Determination of IC_50_

**Figure 6:**
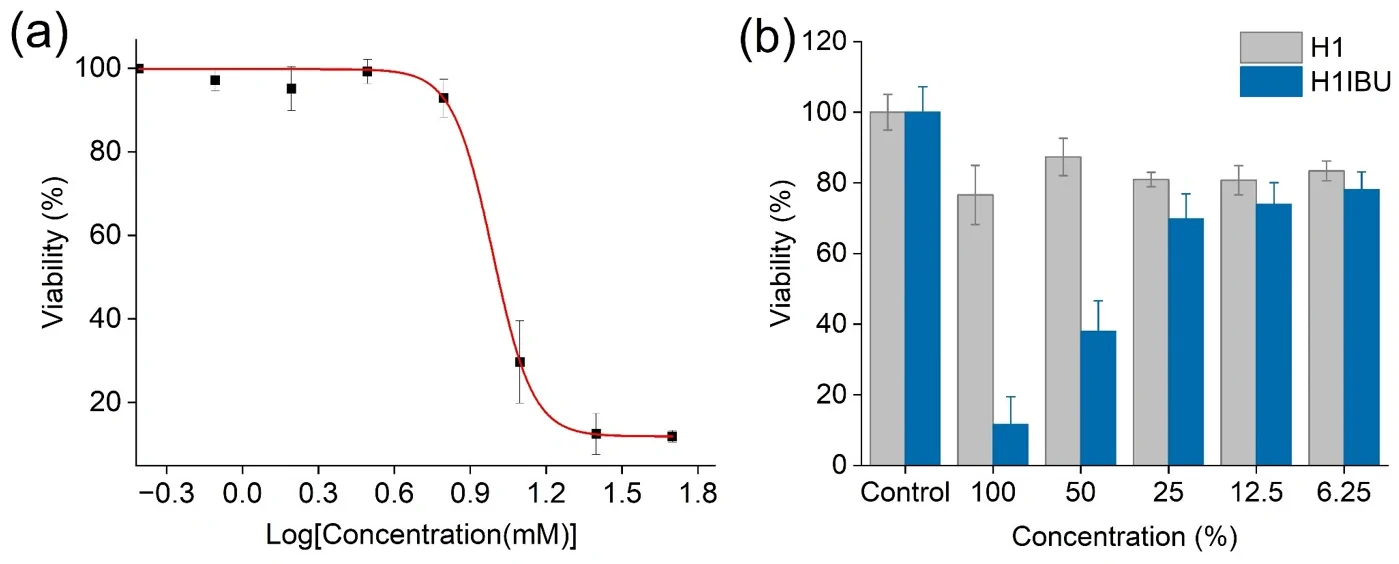
Cytotoxicity assessment of hydrogel extracts in HeLa cells. (**a**) Dose–response curve for the determination of the half-maximal inhibitory concentration (IC_50_ = 9.75 ± 0.66 mM) of pure ibuprofen using a non-linear regression. (**b**) Cell viability evaluated by MTT assay (570 nm) after 72 h of exposure to serial dilutions of drug-free hydrogel (H1) and ibuprofen-loaded hydrogel (H1IBU). Data are presented as mean ± SD (*n* = 3).

Evaluation of ibuprofen cytotoxicity in HeLa cells showed a dose-dependent inhibitory effect, with an IC_50_ of 9.75 mM (Figure 6a). This millimolar potency aligns with published reports describing the concentration range required for NSAIDs to induce cytotoxic responses in neoplastic cell lines [31]. The high goodness-of-fit to the sigmoidal adjustment (R^2^ = 0.999), together with a pronounced Hill slope (*h* = 5.49), suggests a cooperative cytotoxic mechanism, potentially reflecting the rapid saturation of cellular defense pathways beyond a critical concentration threshold.

Although these millimolar concentrations might seem high for systemic treatment, local mucosal delivery uniquely allows such regional drug levels to be achieved safely without systemic toxicity [31]. However, the in vitro antiproliferative response in HeLa cells is an exploratory, secondary observation rather than a proposed standalone therapy for cervical cancer. Instead, the main value of ibuprofen in this setting lies in its ability to control local inflammation, which may offer useful adjuvant benefits alongside conventional therapies [29]. Such an approach could combine local anti-inflammatory effects with complementary cytotoxic activity to enhance the efficacy of conventional chemotherapeutic agents. Previous studies have suggested that such combination strategies may allow lower effective doses and, consequently, reduce systemic adverse effects [108,109,110].

#### 2.7.2. Hydrogel Cytotoxicity Assessment

To evaluate the safety and delivery potential of the synthesized hydrogel formulations, HeLa cells were exposed to H1 and H1IBU extracts for 72 h (Figure 6b). H1 showed high biocompatibility across all tested dilutions, maintaining cell viability above 75% even at the 100% extract concentration. This result indicates that the polymer matrix does not release toxic degradation products or unreacted monomers in quantities sufficient to induce cellular distress.

In contrast, H1IBU exhibited a concentration-dependent cytotoxic response. At 100% extract concentration, cell viability decreased to approximately 12%, indicating that the ibuprofen was successfully released from H1IBU into the culture medium while retaining its biological activity. Progressive dilution of the H1IBU extract (from 100% to 6.25%) gradually recovered cell viability, reaching approximately 78% at 6.25%. This observed cytotoxic effect can be attributed specifically to the released ibuprofen rather than the hydrogel matrix, consistent with established drug-release behaviour [30,111]. Importantly, these findings provide evidence of bioactive drug release from the hydrogel network rather than standalone anticancer efficacy, which would require dedicated mechanistic and in vivo evaluations.

The vaginal physiological environment is expected to play a critical role in the in vivo performance of the hydrogel. Following administration, the formulation is exposed to body temperature (≈37 °C), an acidic vaginal pH (4.0–4.6 in healthy women), continuous vaginal fluid turnover, and mechanical stresses associated with daily activities, all of which influence gelation, rheological behaviour, drug release, and residence time [112,113]. In the present study, the rapid sol-to-gel transition observed at physiological temperatures, together with the marked increase in viscosity, suggests the formulation’s potential for in situ gelation, which could help minimize leakage upon application. Furthermore, the in vitro swelling and gel persistence observed upon incorporating CP940 and HPMC indicated favourable hydration dynamics and physical cohesion—properties desirable for localized vaginal drug delivery [114,115].

Furthermore, the release studies performed in SVF provide a physiologically relevant assessment of the formulation under conditions that closely resemble the healthy vaginal environment. At this pH, ibuprofen exists as a mixture of ionized and non-ionized forms [28,63]. One possible explanation for the sustained-release profile is that a fraction of the drug remained preferentially associated with the hydrophobic PPO domains of PF127 micelles, which act as drug reservoirs. At the same time, the three-dimensional PF127/CP940/HPMC network imposed an additional diffusional barrier to drug transport. The Peppas–Sahlin model supports this interpretation, showing that Fickian diffusion accounted for 69.05% of cumulative drug release, while polymer relaxation contributed 30.95%, indicating that diffusion was the predominant release mechanism. Taken together, these findings demonstrate that the selected polymer combination provides thermoresponsive, in vitro adhesive, and sustained-release properties well suited to the physiological vaginal environment, supporting its potential as a platform for prolonged, localized vaginal drug delivery.

Compared with existing poloxamer-based in situ gels, the present hydrogel addresses distinct physiological and therapeutic constraints. For instance, the injectable PF127/HPMC/CP940 depot developed for anti-tuberculosis therapy [19] gelled at a lower temperature (26 °C), showed markedly lower sol/gel viscosities (238/1700 cP) tailored for needle administration, sustained release over 6–10 days, and was optimized for high biocompatibility. Similarly, the poloxamer/Carbopol binary gel for periodontal quercetin delivery [30] preserved cell viability, gelled at 36 °C, and displayed a gel viscosity (151,798 mPa·s) within the same order of magnitude as our H1 matrix. In contrast, the present vaginal hydrogel was designed to address the particular requirements of localized drug delivery to the vaginal mucosa. It gels at 28 °C and exhibits viscosities that are an order of magnitude higher than those reported for injectable poloxamer/HPMC/Carbopol formulations [19,36]. Furthermore, it operates within a shorter release window of hours rather than days and exerts a targeted antiproliferative effect on HeLa cervical cancer cells.

To our knowledge, the ternary PF127/HPMC/CP940 formulation has not been previously reported for vaginal administration. Beyond the formulation design, this work demonstrates the feasibility of incorporating a poorly water-soluble model drug within this thermosensitive network while preserving its phase-transition temperature, rheological behaviour, and adhesive capacity. Moreover, while most published vaginal thermogels are validated through material characterization or a single biological assay, the present study integrates physicochemical analyses, kinetic release modelling, and quantitative bioassays within a single framework.

The novelty of this platform, therefore, lies in the rational adaptation of this ternary matrix for potential vaginal delivery and an integrated evaluation approach. Together, these foundational findings establish a well-characterized vehicle, laying the essential groundwork for subsequent in vivo tolerance studies, pH-recovery kinetics, and preclinical efficacy assessments before clinical translation. Furthermore, it provides a versatile baseline for exploring a broader spectrum of hydrophobic therapeutic agents for localized vaginal mucosal delivery.

### 2.8. Study Limitations

Although this study provides information on the feasibility of developing thermosensitive hydrogels loaded with ibuprofen and allows characterization of their physicochemical behaviour and in vitro drug release profile under simulated vaginal conditions, several limitations must be considered when interpreting these results. In particular, the findings constitute an initial evaluation of the formulation and, on their own, do not establish its performance, retention, safety, or efficacy in the vaginal environment. The use SVF provides a controlled environment for evaluating the formulation’s behaviour under defined pH and temperature conditions; however, this simplified model does not fully replicate the complexity of the vaginal microenvironment. Factors such as the presence and turnover of mucus, the dynamic interaction between the formulation and the epithelial surface, tissue permeability, and drug distribution and elimination can substantially alter the behaviour of the system. For this reason, the in vitro release profiles obtained in this study should be interpreted primarily as a characterization of ibuprofen release from the polymer matrix, rather than as a direct measure of its availability, permeation, or retention in vaginal tissue.

Furthermore, although the physicochemical properties evaluated allow determination of the fundamental characteristics of the hydrogel and support its further investigation as a vaginal delivery platform, this study did not directly evaluate vaginal retention or the interaction between the formulation and the tissue. Future studies should incorporate ex vivo and in vivo vaginal tissue models to investigate ibuprofen permeation, local retention, formulation residence time, and behaviour under more biologically relevant conditions.

Finally, this study does not establish the local safety of the formulation. Therefore, these results will need to be supplemented with biological assessments to determine cytocompatibility, vaginal irritation, integrity of the epithelial barrier, the inflammatory response, and local tissue compatibility. Taken together, these limitations define the scope of the current results and outline the next steps needed to determine the safety, performance, and translational potential of the thermosensitive hydrogel as a vaginal delivery system for ibuprofen.

## 3. Conclusions

In this work, we successfully developed and characterised a ternary thermosensitive hydrogel platform comprising PF127, HPMC, and CP940 for potential localized vaginal administration, using ibuprofen as a model drug. The formulation showed a constant gelation temperature of 28 °C, high viscosity at physiological temperature, and, upon ibuprofen incorporation, markedly reduced susceptibility to gravitational leakage, supporting its suitability for vaginal application. Physicochemical characterization using FTIR, TGA, DSC, and XRD confirmed the formation of a stable, physically cross-linked polymeric matrix and effective drug incorporation. Thermal and X-ray diffraction analyses indicated that ibuprofen remains molecularly dispersed or in an amorphous state within the network, a condition highly favourable for consistent drug release. Kinetic modelling indicated a sustained-release profile, governed by a combination of Fickian diffusion and polymer chain relaxation (anomalous transport), yielding sustained drug delivery over 24 h without an initial burst.

From a biological perspective, the blank hydrogel exhibited high biocompatibility toward HeLa cells, remaining well within safety thresholds. Concurrently, ibuprofen release from H1IBU induced a concentration-dependent cytotoxic effect, confirming that the hydrogel matrix functions as an effective delivery platform while preserving the drug’s bioactivity. We regard this antiproliferative response as an exploratory, secondary finding rather than evidence of standalone anticancer efficacy. Collectively, these findings demonstrate that this ternary hydrogel platform is a well-characterized, biocompatible vehicle for sustained local drug delivery in the vaginal microenvironment, providing a foundation for further preclinical development.

## 4. Materials and Methods

### 4.1. Materials

PF127 (Pluronic F127^®^, powder, pharmaceutical grade, Sigma-Aldrich, St. Louis, MO, USA), hydroxypropyl methylcellulose (METHOCEL^®^ K100 Premium, hypromellose 2208, USP grade, Sigma-Aldrich, St. Louis, MO, USA), Carbopol^®^ 940 (pharmaceutical grade, Lubrizol Advanced Materials, Cleveland, OH, USA), ibuprofen salt (α-methyl-4-(isobutyl)phenylacetic acid sodium salt, analytical standard, ≥98% purity, Sigma-Aldrich, St. Louis, MO, USA), analytical-grade triethanolamine (TEA; ≥99.0% purity, Sigma-Aldrich, St. Louis, MO, USA), human cervical cancer cell line HeLa (ATCC^®^, CCL-2, American Type Culture Collection, Manassas, VA, USA), Dulbecco’s Modified Eagle Medium (DMEM; high glucose, with L-glutamine, cell culture grade, Sigma-Aldrich, St. Louis, MO, USA), 3-(4,5-dimethylthiazol-2-yl)-2,5-diphenyltetrazolium bromide (MTT; ≥98% purity, Sigma-Aldrich, St. Louis, MO, USA) and Dimethyl sulfoxide (DMSO; sterile-filtered, cell culture grade, ≥99.7% purity, Sigma-Aldrich, St. Louis, MO, USA).

### 4.2. Methods

#### 4.2.1. Preparation of Hydrogel

Thermosensitive hydrogels were prepared according to the method described by Balu et al. [19], with modifications. The final formulation consisted of 17% (*w*/*w*) PF127, 0.1% (*w*/*w*) CP940, 0.3% (*w*/*w*) HPMC, 0.23% (*w*/*w*) ibuprofen, and distilled water up to a total mass of 50 g. Two formulations were prepared: H1 and H1IBU.

Polymer suspensions were prepared individually as follows. PF127 (8.5 g in 30 mL) was hydrated at 4 °C for 3 h. CP940 (0.05 g in 4 mL) was hydrated for 24 h at room temperature and homogenized under magnetic stirring. HPMC (0.15 g) was dissolved under mechanical agitation at 60 °C for 3 min. Separately, an ibuprofen solution was prepared by suspending 114 mg of the drug in 4 mL of distilled water using vortex mixing.

To assemble the hydrogel, CP940, HPMC, and ibuprofen dispersions were sequentially added to the cold PF127 dispersion under continuous magnetic stirring at 4 °C to prevent premature thermal gelation. The mixture was homogenized at 4 °C for 24 h to ensure complete hydration and uniform dispersion of the drug.

Subsequently, analytical-grade TEA was added dropwise until the pH reached 7.0. Based on an estimated drop volume of approximately 0.04 mL when dispensed using a Pasteur pipette, approximately 0.04 mL (one drop) of TEA was required for H1IBU. In contrast, H1 required approximately 0.08 mL (two drops). Because each formulation contained 0.05 g of CP940, these amounts corresponded to approximately 0.9 and 1.8 g of TEA per gram of CP940 for H1IBU and H1, respectively. The formulation was stirred for an additional 10 min to ensure uniform TEA distribution, complete CP940 neutralization, and matrix stabilization. Finally, hydrogels were autoclaved (121 °C, 15 min) and stored in hermetically sealed containers at 4 °C until further characterization. All experiments were performed using hydrogels stored at 4 °C for up to 1 month to ensure integrity.

#### 4.2.2. Physicochemical and Functional Performance Evaluation

**pH evaluation**. Hydrogel pH was measured using a potentiometer (PHS-3C, Shanghai Skill Science Instrument Co., Ltd., Shanghai, China, WMETERS) following 1 min of sample thermal equilibration. All measurements were conducted in triplicate (*n* = 3).

**Dynamic viscosity.** The dynamic viscosity of sterilized hydrogels (autoclaved at 121 °C for 15 min) was determined using a rotational viscometer (ViscoQC 300-R, V-Curve version 1.80, Anton Paar, GmbH, Graz, Austria). Aliquots of approximately 50 mL were transferred to the sample container and equilibrated at the target temperature (25 ± 0.5 °C for storage conditions and 37 ± 0.5 °C for physiological conditions). Measurements were recorded at 50 rpm using a designated spindle for semisolid samples (*n* = 3, mean ± standard deviation (SD)).

**Preparation of SVF.** SVF was prepared as described by Owen and Katz [116], with the pH adjusted to 4.6 or 7.0. Briefly, 3.510 g sodium chloride (NaCl), 1.400 g potassium hydroxide (KOH), 0.222 g calcium hydroxide Ca(OH)_2_, 0.018 g albumin, 2 g lactic acid (C_3_H_6_O_3_), 1 g acetic acid (CH_3_COOH), 0.160 g glycerol (C_3_H_8_O_3_), 0.400 g urea (CH_4_N_2_O) and 5 g of dextrose (C_6_H_12_O_6_) were dissolved in distilled water. The solution was brought to a final volume of 1 L, and the pH was adjusted to 4.6 or 7.0, depending on the test, using hydrochloric acid (HCl) or sodium hydroxide (NaOH) to achieve the desired pH.

**Swelling behaviour**. We evaluated the hydrogel swelling capacity in SVF at 37 °C. To determine the initial dry weight, we transferred approximately 3 g of each formulation to Petri dishes and dehydrated them in a convection oven at 45 °C for 48 h. Mass loss was monitored hourly for the first 12 h, and weight constancy was verified at 24 and 48 h to ensure complete solvent removal. Dried hydrogel samples were immersed in 5 mL of SVF. At designated intervals (hourly during the first 4 h and at 24 h to assess swelling equilibrium, samples were retrieved and carefully blotted with filter paper to remove excess surface liquid without compressing the polymer matrix. They were weighed (*n* = 3, mean ± SD).

**Water retention ratio.** The fluid absorption and retention dynamics of the hydrogels were evaluated using the swelling ratio (*SR*) and the water retention ratio (*WRR*) [117,118,119]. While *SR* reflects the expansion capacity of the polymeric matrix relative to its dry weight, *WRR* quantifies the percentage of retained water over time [117,118,119]. Both parameters were calculated according to Equations (1) and (2):

(1) $$SR=\frac{W_{t}-W_{0}}{W_{0}}$$

(2) $$WRR\;(\%)=\frac{W_{t}}{W_{0}}\times 100$$

where *W_t_* represents the weight of the hydrated hydrogel at time (*t*), and *W*_0_ corresponds to the initial dry weight.

**Gelling temperature and gelation time**. The sol-to-gel transition temperature and gelation time were determined using the tube inversion method [19]. Briefly, 2 mL aliquots of cold hydrogel (4 °C) were transferred into 15 mL glass test tubes and placed in a temperature-controlled water bath. The system was heated from 20 °C at a rate of 1 °C min^−1^. At each 1 °C increment, the tube was tilted to 90° and held for 30 s. Gelation temperature was recorded as the lowest temperature at which no fluid flow or meniscus movement occurred upon inversion. Simultaneously, gelation time was defined as the total time required to achieve complete gelation (*n* = 3, mean ± SD).

**Gel persistence capacity**. We evaluated hydrogel matrix persistence according to a modified protocol described by Patel et al. [77]. Formulations (1 mL) were transferred into glass vials containing 2 mL of SVF and incubated at 37 °C under continuous orbital shaking at 50 rpm (Shaker Incubator THZ-100, Luzeren, Shanghai, China). Samples were visually monitored, and persistence time was defined as the period required for complete matrix erosion or disintegration (*n* = 3, mean ± SD).

**Spreadability.** Hydrogel spreadability was determined using a parallel-plate compression method adapted from Afzaal et al. [78]. Briefly, 1 g of sample was placed at the center of a glass Petri dish (90 mm diameter × 20 mm height), covered with an identical upper dish, and thermally equilibrated at 37 °C. A 100 g weight was applied to the top plate for 1 min to allow uniform spreading of the formulation. After removing the weight, the resulting spreading diameter was measured in millimetres (*n* = 3; mean ± SD).

**In vitro leakage evaluation**. Leakage behaviour of hydrogel formulations was evaluated using an adapted protocol [78]. Briefly, 2 g of formulation was mixed with 0.75 mL of SVF and incubated at 37 ± 1 °C for 24 h to simulate post-administration hydration. Model mucosal surfaces were prepared by uniformly coating glass slides with a 1.5% (*w*/*v*) agar gel layer. Next, 100 mg of each SVF-conditioned formulation was applied to one end of the agar-coated slide. The slides were positioned vertically (90°) in a glass beaker containing 3–5 mL of distilled water and incubated at 37 °C for 1 h under agitation at 50 rpm. The leakage distance of the hydrogel along the agar surface was measured in millimeters (*n* = 3, mean ± SD).

**In vitro adhesion evaluation**. We evaluated the adhesive properties of the hydrogel formulations using a modified protocol adapted from Afzaal et al. [78]. Glass microscope slides were coated with a 1.5% (*w*/*v*) agar gel to model the mucosal surface. Hydrogel aliquots (100 mg) were applied to one end of each agar-coated slide. The loaded slides were vertically immersed (90°) in glass beakers filled with SVF. The assemblies were incubated at 37 ± 1 °C under continuous agitation at 50 rpm in an orbital shaker. Adhesion time was defined as the duration the hydrogel remained attached to the agar surface before detaching (*n* = 3, mean ± SD).

#### 4.2.3. Structural and Thermal Characterization

**Fourier transform infrared spectroscopy**. The FTIR spectra were acquired using a PerkinElmer Frontier FTIR spectrometer (Waltham, MA, USA) equipped with a universal attenuated total reflectance (ATR) accessory. Spectra were recorded over 4000–650 cm^−1^ at a spectral resolution of 4 cm^−1^, accumulating 15 co-added scans per sample.

**Thermal analyses.** We characterized the thermal behaviour and phase transitions of pure polymers and hydrogel formulations using thermogravimetric analysis (TGA) and differential scanning calorimetry (DSC). TGA was performed on a Mettler Toledo TGA/DSC 1 Star system under a nitrogen purge (20 mL min^−1^) at a heating rate of 10 °C min^−1^ over a temperature range of 30–600 °C. DSC measurements were conducted using a PerkinElmer DSC 8000 (PerkinElmer, Waltham, MA, USA). Samples of 4–6 mg were subjected to heating and cooling cycles at 10 °C min^−1^ under a nitrogen flow of 20 mL min^−1^.

**Water loss**. We evaluated water loss and evaporation kinetics by isothermal thermogravimetric analysis. Hydrogel samples (70–77 mg) were loaded into standard TGA pans and maintained at 37 °C under isothermal conditions for 4 h to monitor water evaporation via continuous mass-loss tracking.

**X-ray diffraction analyses**. Crystallinity profiles were collected on a PANalytical Empyrean diffractometer (Panalytical, Malvern, UK) operating with Cu-Kα radiation (λ = 0.154 nm). Formulations mounted on a flat glass sample holder were scanned from 2*θ* = 5° to 90° (*θ*/*θ* geometry) with a step size of 0.026° and a 30 s dwell time per step.

#### 4.2.4. In Vitro Drug Release Study and Release Kinetics

**In vitro release testing**. The in vitro release profile of ibuprofen from the hydrogel matrix was evaluated using the dialysis membrane diffusion method. Briefly, 1 g of H1IBU was placed into a dialysis bag, securely sealed at both ends, and immersed in 50 mL centrifuge tubes containing 10 mL of SVF. The study was conducted under sink conditions at 37 ± 0.5 °C with continuous agitation at 100 rpm using an orbital shaker. Aliquots (2 mL) were withdrawn at predetermined time intervals (every 15 min during the first hour, every 30 min from 1 to 6 h, hourly from 6 to 12 h, and at 24 h) and immediately replaced with an equal volume of fresh prewarmed SVF (37 °C) to maintain a constant dissolution volume.

Ibuprofen released was quantified by UV–visible spectrophotometry using a GENESYS 10 UV spectrophotometer (Thermo Scientific, Madison, WI, USA). Release profiles were constructed by plotting the cumulative percentage of ibuprofen released (*M_t_*/*M∞*, %) against time (*t*, min). Absorbance was measured at the maximum absorption wavelength (λ_max_ = 222 nm). Cumulative drug release was corrected for volume and calculated based on the total theoretical drug incorporated into the matrix. Because the hydrogel was prepared by directly incorporating dissolved ibuprofen into the polymeric network without filtration or washing steps, no drug loss was assumed during formulation.

**Method selectivity**. To verify analytical specificity and confirm the absence of interference from formulation excipients or SVF components, UV–Vis absorption spectra (200–300 nm) of pure ibuprofen, blank hydrogel, and SVF were recorded. Spectral comparison confirmed no overlapping absorption bands at 222 nm, demonstrating selective detection and quantification of ibuprofen throughout the release assay.

**Release kinetics modelling**. To elucidate the transport mechanisms governing drug release, we fitted experimental data up to 50% cumulative release to the Peppas–Sahlin equation using Microsoft Excel^®^ Solver. This model discriminates between Fickian diffusion and polymer chain relaxation within thermoresponsive networks (Equation (3)).

(3) $$\frac{M_{t}}{M\infty }=K_{f}t^{m}+K_{r}t^{2m}$$

where $\frac{M_{t}}{M\infty }$ represents fractional drug release at time *t*, *K_f_* and *K_r_* are the Fickian diffusion and relaxation rate constants, and *m* is the Fickian kinetic exponent (bound identically to the planar geometry *n* parameter).

**Cell culture**. HeLa human cervical carcinoma cells were cultured in Dulbecco’s Modified Eagle Medium (DMEM; Sigma-Aldrich, St. Louis, MO, USA) supplemented with 10% fetal bovine serum (FBS; Gibco, Waltham, MA, USA) and 1X PSN antibiotics mixture (Sigma-Aldrich). Monolayers were maintained at 37 °C in a humid atmosphere containing 5% CO2. At 70–80% confluence, cells were washed with PBS, detached using 0.25% trypsin, and neutralized with complete medium. Following centrifugation, the cell pellet was resuspended in fresh medium for experimental seeding.

**Determination of IC_50_.** Cytotoxicity was evaluated via 3-(4,5-dimethylthiazol-2-yl)-2,5-difeniltetrazolium bromide (MTT) assay. HeLa cells were seeded into 96-well plates (~1 × 10^4^ cells/well) and allowed to adhere for 24 h at 37 °C. Then the culture medium was replaced with fresh medium containing ibuprofen at concentrations across a gradient (0.08, 0.1, 0.35, 0.7, 1.4, 2.8, 5.7, and 11.4 mg/mL) for 24 h, alongside untreated controls. After exposure, 10 µL of MTT solution (5 mg/mL in PBS) was added to each well, and the plate was incubated for 3 h at 37 °C. Supernatants were removed, and the resulting formazan precipitates were dissolved in 100 μL of DMSO. Absorbance was recorded at 570 nm using a microplate reader (Bio-Rad, Hercules, CA, USA). Cell viability is expressed relative to untreated control cells (100% viability) according to Equation (4):

(4) $$Cell\;viability\;\left(\%\right)=\frac{{Sample}_{abs}}{{Control}_{abs}}\times 100$$

The half-maximal inhibitory concentration (IC_50_) was derived from dose–response curves using non-linear regression analysis in OriginPro software (version 2021; OriginLab Corporation, Northampton, MA, USA).

**Hydrogel cytotoxicity assessment**. Hydrogel extracts—both drug-free and ibuprofen-loaded (22.826 mg/mL)—were prepared by incubating 500 mg of sterile sample in 5 mL of DMEM for 72 h at 37 °C under agitation (50 rpm). Supernatants were centrifuged (1000 rpm, 5 min) and filtered (0.45 μm) to remove particulate debris. HeLa cells were seeded into 96-well plates (1 × 10^4^ cells/well) and exposed to five extract concentrations prepared via serial twofold dilutions (100%, 50%, 25%, 12.5%, and 6.25%) for 72 h. Cell viability was determined using the MTT assay by measuring absorbance at 570 nm.

## Figures and Tables

**Table 1:** Physicochemical properties of the sterilized drug-free (H1) and ibuprofen-loaded (H1IBU) thermosensitive hydrogels.

Hydrogel	pH (Dimensionless)	Viscosity (mPa·s)	Temperature (°C)	Swelling Ratio (g/g)	Water Retention (%)
H1	7.14 ± 0.114 ^a^	150,833 ± 41,135 ^a^	25	2.58 ± 0.16 ^a^	353.42 ± 22.8 ^a^
		2,603,333 ± 223,681 ^b^	37		
H1IBU	7.34 ± 0.114 ^a^	143,233 ± 4485 ^a^	25	0.75 ± 0.026 ^b^	182.77 ± 15.33 ^b^
		2,112,333 ± 270,601 ^c^	37		

Viscosity measurements at 25 °C and 37 °C were performed at shear rates of 20 rpm and 0.2 rpm, respectively, while temperature was maintained as a controlled experimental condition. For each parameter, values bearing different superscript letters (a–c) denote statistically significant differences (*p* < 0.05) as determined by two-way ANOVA followed by Tukey’s post hoc test. Data expressed as mean ± SD (*n* = 3).

**Table 2:** Physicochemical, rheological, and performance parameters of H1 and H1IBU hydrogel systems.

Hydrogel	Gelation Temperature (°C)	pH	Gelation Time (s)	Spreadability (mm)	Gel Persistence Capacity (min)	Leakage (mm)	Adhesion (min)
H1	28	4.6	43.33 ± 5.77 ^a^	-	178.5 ± 44.5 ^a^	51.67 ± 5.77 ^a^	24 ± 5.29 ^a^
	28	7.0	25 ± 5 ^b^	14 ± 1.73 ^a^	62 ± 14.93 ^b^	-	-
H1IBU	28	4.6	38.33 ± 2.88 ^a^	-	76.67 ± 17.55 ^b^	10.33 ± 0.58 ***	8.77 ± 1.08 ^b^
	28	7.0	16.66 ± 2.88 ^b^	14.66 ± 0.577 ^a^	56 ± 9.64 ^b^	-	-

Gelation temperature (28 °C) and pH (4.6 and 7.0, dimensionless) represent controlled experimental conditions. Within each parameter, different superscript letters (a, b) indicate statistically significant differences (*p* < 0.05), whereas asterisks (***) indicate highly significant differences (*p* < 0.001) determined by Student’s *t*-test. Data are expressed as mean ± SD (*n* = 3), except for gelation temperature and pH, which were held constant.

**Table 3:** Thermal properties of drug-free (H1) and ibuprofen-loaded (H1IBU) hydrogels and their individual components.

Polymer		TGA ^a^		DSC ^b^			
				Cooling		Second Heating	
	*T*_d_ (°C)	^max^*T*_d_ (°C)	*R*_w_ (%)	*T*_c_ (°C)	Δ*H*_c_ (J·g^−1^)	*T*_m_ (°C)	Δ*H*_m_ (J·g^−1^)
PF127	366	392	0	30	−108	57	110
CP940	254	117/509	12	-	-	133	2
HPMC	323	362	7	-	-	-	-
Ibuprofen	159	467	14	34	−0.3	65	0.3
H1	370	393	2	32	−98	57	98
H1IBU	357	403	2	30	−105	56	99

^a^ Onset decomposition temperature corresponding to 10% weight loss (*T*_d_), temperature of maximum decomposition rate (^max^*T*_d_), and remaining residual weight (*R*_w_) determined by TGA after heating at 600 °C. ^b^ Crystallization temperature (*T*_c_), crystallization enthalpy (Δ*H*_c_), melting temperature (*T*_m_), and melting enthalpy (Δ*H*_m_) determined by DSC.

**Table 4:** Kinetic parameters derived from the Peppas–Sahlin model fitting for ibuprofen release from H1IBU hydrogel.

Parameter	Description	Value	Units
*k*_1_	Fickian diffusional rate constant	0.0748	min^−m^
*k*_2_	Polymer relaxation/erosion rate constant	0.0061	min^−2m^
*m*	Diffusional exponent	0.2364	—
*k*_1_/*k*_2_	Ratio of kinetic constants (Fickian/Relaxation)	12.30	—
SSE	Sum of Squared Errors	2.7258	—

Kinetic parameters were obtained by non-linear regression using the Generalized Reduced Gradient (GRG) algorithm in Microsoft Excel Solver. The optimization minimized the sum of squared errors (SSE) objective function under the physical constraints (*k*_1_ ≥ 0, *k*_2_ ≥ 0, and *m* > 0).

## Data Availability

The original contributions presented in the study are included in the article/Supplementary Materials. Further inquiries can be directed to the corresponding authors.

## Supplementary Materials

The following supporting information can be downloaded at https://www.mdpi.com/article/10.3390/gels12090807/s1. Figure S1. FTIR spectra of individual raw polymers (PF127, HPMC, and CP940) compared to the drug-free ternary hydrogel (H1); Figure S2. Thermogravimetric (TGA) and derivative thermogravimetric (DTG) curves of hydrogel formulations and starting raw materials; Figure S3. Differential scanning calorimetry (DSC) thermograms of hydrogel formulations and individual components across thermal cycles.
