# Supplementary material for: Poloxamer/HPMC/Carbopol-Based Thermosensitive Hydrogel Loaded with Ibuprofen for Potential Vaginal Drug Release
Source: Gels. 2026 Sep 3;12(9):807. doi: 10.3390/gels12090807 (PMC13606056; doi:10.3390/gels12090807)
Supplement: Supplementary file 1 [file gels-12-00807-s001.zip › gels-4464241-supplementary.pdf]

Poloxamer/HPMC/Carbopol-based thermosensitive hydrogel loaded with ibuprofen for potential vaginal drug release

Gladys Arline Politrón Zepeda[1], Ernesto Tinajero-Díaz[2,*], Antxon Martínez de Ilarduya[2], Rogelio Rodríguez Rodríguez[3], Gregorio Guadalupe Carbajal Arízaga[4], Aldo Corona Escalera[1], Nathaly Vasquez Martínez[5], Moisés Martínez Velázquez[1,*] and Zaira Yunuen García Carvajal[1,*]

[1] Biotecnología Médica y Farmacéutica, Centro de Investigación y Asistencia en Tecnología y Diseño del Estado de Jalisco A.C., (CIATEJ), Normalistas No. 800, Guadalajara, 44270, Jalisco, México; glpolitron_al@ciatej.edu.mx; mmartinez@ciatej.mx; zgarcia@ciatej.mx
[2] Departament d'Enginyeria Química, Universitat Politècnica de Catalunya, Barcelona School of Industrial Engineering (ETSEIB), Diagonal 647, Barcelona, Spain; ernesto.tinajero@upc.edu; antxon.martinez.de.ilarduia@upc.edu
[3] Departamento de Ciencias Naturales y Exactas, Centro Universitario de los Valles (CUVALLES), Universidad de Guadalajara, Carretera Guadalajara-Ameca Km. 45.5, Ameca 46600, Jalisco, México; rogelio.rodriguez4085@academicos.udg.mx
[4] Departamento de Química, Universidad de Guadalajara, CUCEI, 44430 Guadalajara, Jalisco, México; gregorio.carbajal@red.cucei.udg.mx
[5] Departamento de Bioquímica, Facultad de Medicina, Universidad Nacional Autónoma de México, Ciudad de México, 04510, México; nathaly@bq.unam.mx
* Correspondence: ernesto.tinajero@upc.edu (E.T-D); mmartinez@ciatej.mx (MMV); zgarcia@ciatej.mx (ZY GC)

Supplementary material

Contents

Figure S1. Comparative spectra of the pure polymers: PF127, HPMC and CP 940 vs. the blank ternary hydrogel H1. 2

Figure S2. TGA traces of PF127, CP940, HPMC and the H1 hydrogel. 3

Figure S3. DSC curves of PF127, HPMC, CP940, and the H1 hydrogel 4

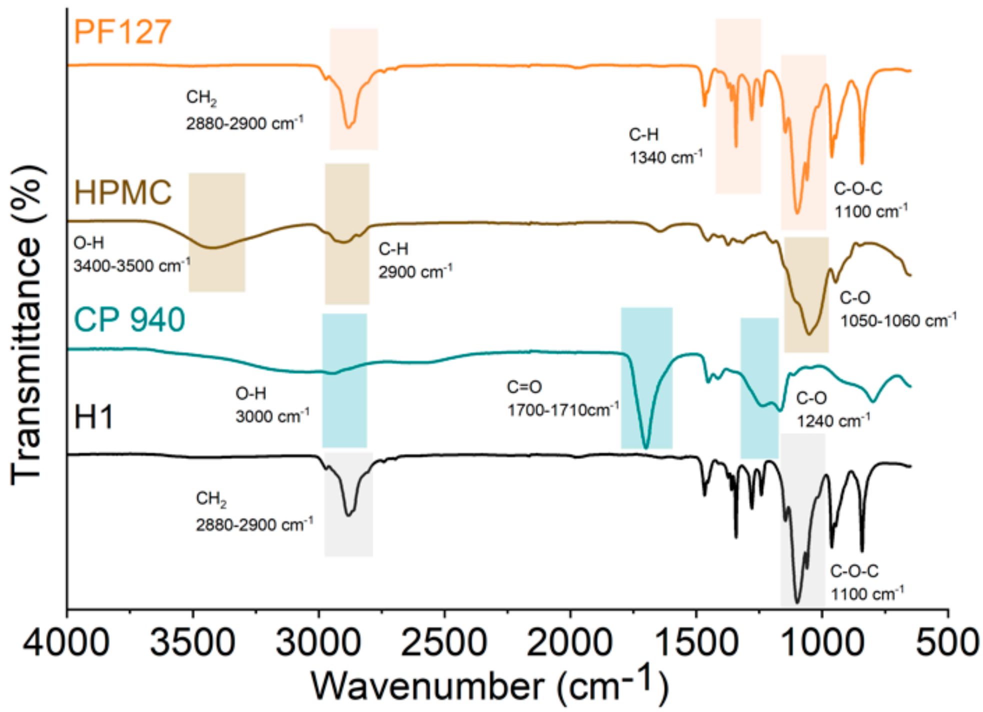

Figure S1. FTIR spectra of individual raw polymers (PF127, HPMC, and CP940) compared to the drug-free ternary hydrogel (H1). Shaded regions highlight characteristic vibrational bands corresponding to key functional groups.

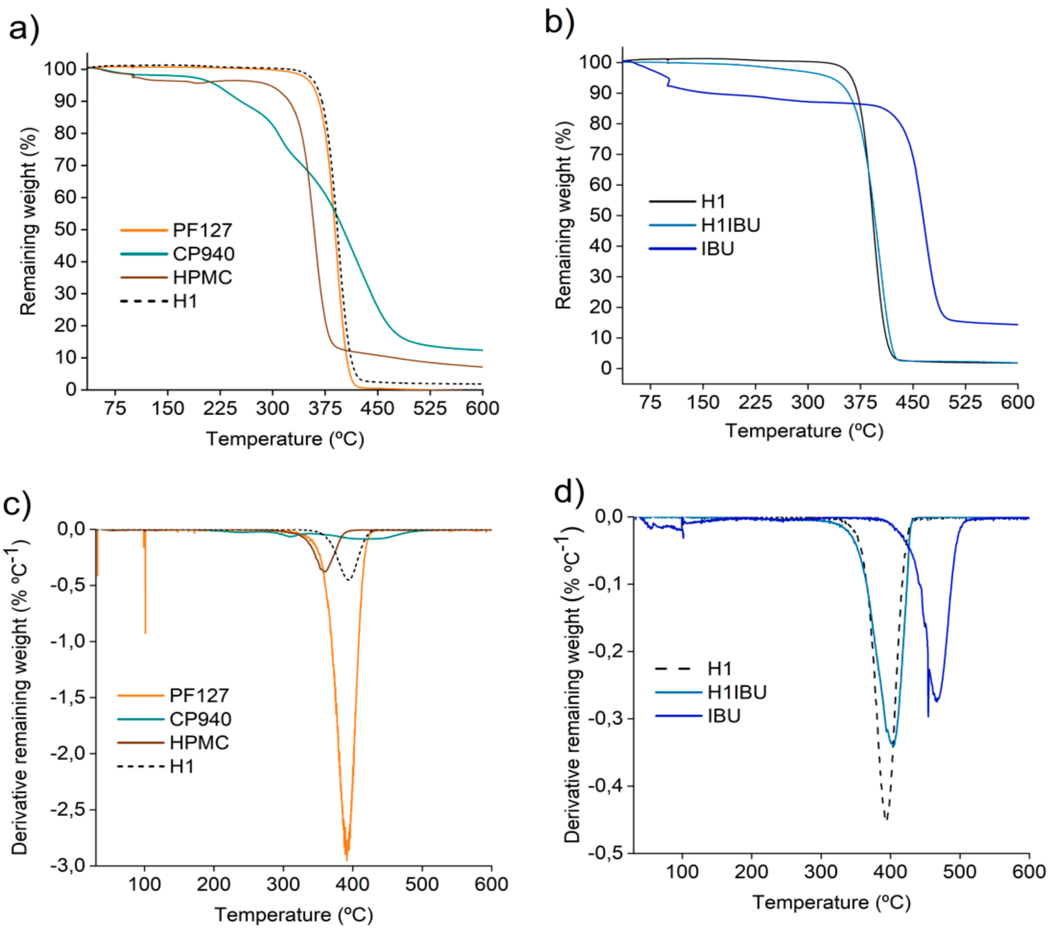

Figure S2. Thermogravimetric (TGA) and derivative thermogravimetric (DTG) curves of hydrogel formulations and starting raw materials**.** (a) TGA thermograms of individual polymers (PF127, CP940, and HPMC) compared to the drug-free hydrogel (H1). (b) TGA thermograms of H1, ibuprofen-loaded hydrogel (H1IBU), and pure ibuprofen (IBU). (c) DTG curves of individual polymer and H1 hydrogel. (d) DTG curves of H1, H1IBU, and pure IBU, illustrating the thermal degradation stages of the system.

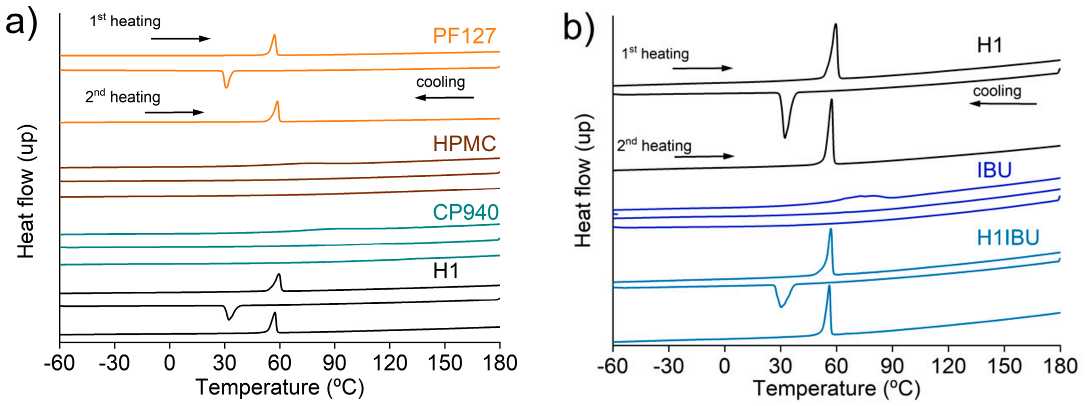

Figure S3. Differential scanning calorimetry (DSC) thermograms of hydrogel formulations and individual components across thermal cycles**.** (a) DSC curves of starting polymers (PF127, HPMC, and CP940) compared to the drug-free hydrogel (H1) during the first heating, cooling, and second heating cycles. (b) DSC thermograms of H1, pure ibuprofen (IBU), and ibuprofen-loaded hydrogel (H1IBU).
